# Simultaneous cotargeting of ATR and RNA Polymerase I transcription demonstrates synergistic antileukemic effects on acute myeloid leukemia

**DOI:** 10.1038/s41392-019-0076-3

**Published:** 2019-11-01

**Authors:** Tingting Wang, Margaret Shatara, Fangbing Liu, Tristan Knight, Holly Edwards, Guan Wang, Hai Lin, Yue Wang, Jeffrey W. Taub, Yubin Ge

**Affiliations:** 1 National Engineering Laboratory for AIDS Vaccine, Key Laboratory for Molecular Enzymology and Engineering, the Ministry of Education, School of Life Sciences, Jilin University, 130021 Changchun, People’s Republic of China; 2 Division of Pediatric Hematology/Oncology, Department of Pediatrics, Children’s Hospital of Michigan, Detroit, MI 48201 USA; 3 Department of Pediatrics, Wayne State University School of Medicine, Detroit, MI 48201 USA; 4 Department of Oncology, Wayne State University School of Medicine, Detroit, MI 48201 USA; 5 Molecular Therapeutics Program, Barbara Ann Karmanos Cancer Institute, Wayne State University School of Medicine, Detroit, MI 48201 USA; 6 Department of Hematology and Oncology, The First Hospital of Jilin University, 130021 Changchun, People’s Republic of China; 7 Department of Pediatric Hematology and Oncology, The First Hospital of Jilin University, 130021 Changchun, People’s Republic of China

**Keywords:** Haematological cancer, Drug development

**Dear Editor,**

Continued development of novel therapeutic agents is critical to improve the survival of patients with acute myeloid leukemia (AML). RNA Polymerase I (Pol I)-mediated transcription and ribosomal biogenesis become dysregulated, thereby allowing synthesis of necessary substrates to support uncontrolled cancer cell proliferation^1^. The Pol I transcription rate is higher in AML cells than nonleukemic myeloid precursors^2^, suggesting Pol I transcription as a therapeutic target for AML. CX-5461 is a potent Pol I transcription inhibitor and stabilizer of the DNA G-quadruplex structure, which causes G2/M-phase arrest via the ATR (ataxia telangiectasia and Rad3-related protein)-mediated DNA damage response (DDR)^3–5^. Therefore, we hypothesized that the ATR-selective inhibitor AZD6738 would synergistically enhance CX-5461-induced cell death via abolishment of the ATR-mediated DDR.

To begin to test our hypothesis, we evaluated apoptosis induction by CX-5461 in AML cell lines. Consistent with previous reports, CX-5461 induced apoptosis at least partially through the intrinsic apoptotic pathway (Fig. S1a–g) and was independent of *TP53* status (Fig. S1h)^6^. Compared with monotherapy, combination treatment of AML cell lines with CX-5461 and AZD6738 for 48 h induced significantly increased apoptosis, as reflected by enhanced Annexin V positivity and substantially increased caspase 3 and PARP cleavage (Figs. 1a, b and S2). Synergistic antileukemic interaction was indicated by combination index (CI) values of <0.77. The combination significantly induced apoptosis within 8 h of drug exposure, exceeding that induced by monotherapy in U937 cells (Fig. 1c). CX-5461 treatment for 24 h caused substantial G2/M-phase arrest (Fig. 1d), while AZD6738 did not appear to induce a major effect. The combination treatment prevented CX-5461-induced G2/M-phase arrest and substantially increased the population of sub-G1 cells (dead cells). Both monotherapy and combination therapy with AZD6738 downregulated CHK-1 protein expression. Combination treatment decreased phosphorylated CDC25C (p-CDC25C) 8–12 h after treatment initiation. CX-5461 caused a time-dependent increase in phosphorylated CDK1 (p-CDK1), which was abolished by combination treatment (Fig. 1e). Similar results were obtained in CTS cells (Fig. S3a–c). In addition, treatment with CX-5461 plus the CHK-1-selective inhibitor LY2603618 abolished CX-5461-induced G2/M-phase arrest and synergistically induced cell death (Fig. S3d and e). Taken together, these findings confirm that AZD6738 synergizes with CX-5461 via abolishment of the G2/M cell cycle checkpoint arrest.

**Fig. 1:**
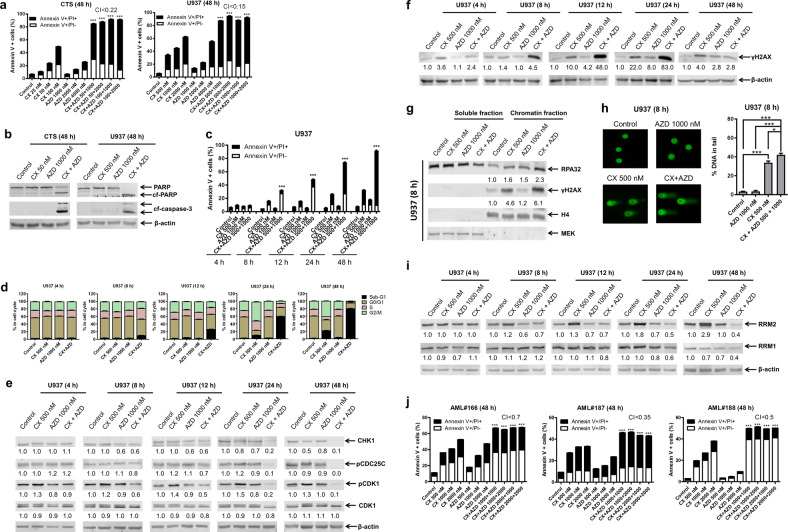
The AML cell lines CTS and U937 and primary AML patient samples were treated with CX-5461 (CX) or AZD6738 (AZD), alone or in combination, for up to 48 h. **a**, **c**, **j** Treated cells were subjected to Annexin V-FITC/PI staining and flow cytometry analyses. **b**, **e**, **f**, **i** Western blots of whole-cell lysates are shown. Fold changes as determined by densitometry with normalization to β-actin, are displayed below each blot. **d** Cell cycle progression was determined by propidium iodide staining and flow cytometry analyses. **g** Chromatin-bound and soluble fractions of RPA32 and γH2AX were analyzed by Western blotting. Fold changes as determined by densitometry, were normalized to histone H4. **h** Representative visualizations of alkaline comet assays are shown (left panel). The results are plotted as the median percentage of DNA in each comet “tail” of four replicates ± SEM (right panel). * indicates *p* < 0.01 and *** indicates *p* < 0.001 (paired two-sample *t* test). CI: combination index, as determined by using CompuSyn software; cf-caspase 3: cleaved caspase 3; cf-PARP: cleaved PARP

Next, we evaluated the effects of these two agents on DNA damage in U937 cells. CX-5461 treatment induced γH2AX at 4 h, while enhancement of γH2AX by the combination was noted starting at 8 h. Within 12 h, AZD6738 increased γH2AX (Fig. 1f). An increase in chromatin-bound RPA32 and γH2AX, indicative of DNA replication stress and damage^7^, was detected after 8 h of combined treatment compared with single-drug treatment (Fig. 1g). The alkaline comet assay results showed a significant increase in the percentage of DNA present in the comet “tail” under combination treatment (Fig. 1h). Similar results were obtained in CTS cells (Fig. S4a–c). Taken together, these results show that CX-5461 and AZD6738 cooperatively induce DNA replication stress and damage in AML cells.

We previously demonstrated that the ATR inhibitor AZ20 causes downregulation of ribonucleotide reductase (RR, a key enzyme in the synthesis of dNDPs) subunits M1 (RRM1) and M2 (RRM2)^8^. Interestingly, CX-5461 treatment increased RRM2 protein expression 8 h post drug treatment, and this increase was abolished by the addition of AZD6738 (Fig. 1i). The RRM1 protein level was largely unchanged, though its downregulation at 24 h was likely due to cell death. Treatment with the RR inhibitor hydroxyurea (HU) significantly enhanced CX-5461-induced cell death (Fig. S4e). Similar results were obtained in CTS cells (Fig. S4d and e). These results show that downregulation of RRM2 likely plays an important role in the synergy between CX-5461 and AZD6738.

Primary AML patient samples (Table S1 shows the patient characteristics) were significantly more sensitive to CX-5461 than normal human peripheral blood mononuclear cells (PBMCs; *p* = 0.007, paired two-sample *t* test), as measured by MTT assays. The AZD6738 IC_50_s of the patient samples showed substantial overlap with those of the healthy controls (*p* = 0.217, paired two-sample *t* test; Fig. S5a, right panel). MTT assays and standard isobologram analyses revealed substantial synergy between CX-5461 and AZD6738 in 10 primary AML patient samples ex vivo (Fig. S5b), which was further confirmed via Annexin V/PI staining and flow cytometry analyses of primary samples from three AML patients for whom adequate blasts were available (Fig. 1j). The combination also showed a synergistic effect in three normal PBMC samples, raising concerns about its potential toxicity (Fig. S5c). However, the sensitivity of primary AML cells greatly exceeded that of normal PBMCs, implying the existence of a therapeutic window.

In summary, our results show that CX-5461 induces DNA damage and ATR activation. ATR has been reported to suppress DNA damage by promoting RRM2 expression^7^. Thus, activation of ATR upregulates RRM2 to aid in the repair of damaged DNA. Therefore, ATR inhibition abolishes the G2/M cell cycle arrest and prevents RRM2 upregulation, decreasing dNTP pools and resulting in the accumulation of damaged DNA and cell death. Our findings support further investigation into the efficacy of CX-5461 in combination with AZD6738 for the treatment of AML.

## Supplementary information

Supplemental File
