# Supplementary material for: Simultaneous cotargeting of ATR and RNA Polymerase I transcription demonstrates synergistic antileukemic effects on acute myeloid leukemia
Source: Signal Transduct Target Ther. 2019 Nov 1;4:44. doi: 10.1038/s41392-019-0076-3 (PMC6823485; doi:10.1038/s41392-019-0076-3)
Supplement: Supplementary file 1 — Supplemental File [file 41392_2019_76_MOESM1_ESM.pdf]

Supplementary Materials for

Simultaneous co-targeting of ATR and RNA Polymerase I transcription demonstrates synergistic antileukemic effects on acute myeloid leukemia

Tingting Wang, Margaret Shatara, Fangbing Liu, Tristan Knight, Holly Edwards, Guan Wang, Hai Lin, Yue Wang, Jeffrey W. Taub*, and Yubin Ge*

Correspondence to: gey@karmanos.org or jtaub@med.wayne.edu

This PDF file includes:

Materials and Methods
References for "Material and Methods" section
Figures. S1 to S5
Table S1

Materials and Methods

Drugs

CX-5461, AZD6738, LY2603618, and hydroxyurea (HU) were purchased from Selleck Chemicals (Houston, TX, USA).

Cell Culture

U937, HL-60, MV4-11, and THP-1 cell lines were purchased from the American Type Culture Collection (Manassas, VA, USA). CMS, CMY, and CTS were gifts from Dr. A Fuse from the National Institute of Infectious Diseases, Tokyo, Japan. MOLM-13 was purchased from AddexBio (San Diego, CA, USA). ML-2, NB-4, OCI-AML3 was purchased from the German Collection of Microorganisms and Cell Cultures (DSMZ, Braunschweig, Germany). The cell lines were cultured in RPMI 1640 media with 10-20% fetal bovine serum (Thermo Fisher Scientific, Rockford, IL, USA) and 2 mM L-glutamine, plus 100 U/mL penicillin and 100 μg/mL streptomycin, in a 37 °C humidified atmosphere containing 5% CO2/95% air, as previously described.[1, 2] Cell lines were tested monthly for the presence of mycoplasma utilizing the PCR method as described by Uphoff and Drexler.[3] All cell lines were authenticated in 2017 at the Genomics Core at Karmanos Cancer Institute using the PowerPlex® 16 System from Promega (Madison, WI, USA). Diagnostic AML blast samples derived from patients were purified by standard Ficoll-Hypaque density centrifugation, then cultured in RPMI 1640 with 20% fetal bovine serum (Thermo Fisher Scientific), ITS Solution (Sigma-Aldrich, St Louis, MO, USA) and 20% supernatant of the 5637 bladder cancer cell line (as a source of granulocyte-macrophage colony-stimulating factor, granulocyte colony-stimulating factor, interleukin-1 beta, macrophage colony-stimulating factor and stem cell factor).[1, 4]

Clinical samples

AML blast samples were obtained from patients at initial diagnosis or at relapse via the First Hospital of Jilin University, Changchun, China, following written informed consent as required by the Declaration of Helsinki. This study was approved by and carried out in accordance with the guidelines as set forth by the Human Ethics Committee of the First Hospital of Jilin University. All clinical samples were screened for the presence of gene mutations by PCR amplification and automated DNA sequencing, and fusion genes by real-time RT-PCR, as previously described.[1, 5] Patient characteristics are displayed in Table S1. AML blast samples were purified via standard Ficoll-Hypaque density centrifugation and cultured, as described previously.[1, 5] Normal bone marrow mononuclear cells (BMMNCs) were purchased from Lonza (Walkersville, MD, USA).

Annexin V/PI staining and flow cytometry analysis

Following treatment with CX-5461 and AZD6738, LY2603618, or hydroxyurea, alone or in combination, for up to 48 hours, AML cells were subjected to flow cytometry analysis utilizing the Annexin V-fluorescein isothiocyanate (FITC)/propidium iodide (PI) apoptosis Kit (Beckman Coulter; Brea, CA, USA), as previously described.[6, 7] Experiments utilizing AML cell lines were performed three times in triplicate, independently, and experiments using primary patient samples were performed once in triplicate due to limited sample, as selected on the basis of adequate sample available for the assay. Apoptotic events are displayed as the mean percentage of Annexin V positive/PI negative (early apoptotic) and Annexin V positive/PI positive (late apoptotic and/or dead) cells ± the standard error from one representative experiment. The extent and direction of antileukemic interaction was determined by calculating the combination index (CI) values using

CompuSyn software (Combosyn Inc., Paramus, NJ, USA). CI < 1, CI = 1 and CI > 1 indicate synergistic, additive, and antagonistic effects, respectively.[8, 9]

Cell cycle progression

Cells were treated with CX-5461 and AZD6738 or LY260318, alone or in combination, for up to 48 h. The cells were fixed with ice-cold ethanol and stained using PI. Cell cycle progression was determined by flow cytometry analyses as previously described.[5]

Western Blot Analysis

Cells were lysed in Tris buffer (10 nM, pH 8.0) containing protease and phosphatase inhibitors (Roche Diagnostics, Indianapolis, IN, USA). Whole cell lysates were subjected to SDS-polyacrylamide gel electrophoresis, electrophoretically transferred onto polyvinylidene difluoride (PVDF) membranes (Thermo Fisher Scientific) and immunoblotted with anti-PARP, -Bax, -CHK1, -β-actin (Proteintech, Chicago, IL), -phosphorylated CDC25C, -MEK, -cleaved caspase-3 (9661, designated -cf caspase-3, Cell Signaling Technology, Danvers, MA, USA), -Bak, -RRM1, -RRM2, -phosphorylated CDK1, -CDK1, (Abcam, Cambridge, MA, USA), -Histone H4 (Upstate Biotechnology, Lake Placid, NY, USA), -γH2AX (Millipore, Billerica, MA, USA), or -RPA32 (Thermo Fisher Scientific), as previously described.[1, 10, 11] Immunoreactive proteins were visualized using the Odyssey Infrared Imaging System (Li-Cor, Lincoln, NE, USA), as described by the manufacturer. Western blots were repeated at least three times, and a single representative blot is shown. Densitometry measurements were made using Odyssey V3.0 (Li-Cor), normalized to β-actin, and calculated as the fold-change in comparison to the corresponding no-drug treatment control (set as '1').

shRNA Knockdown

The pMD-VSV-G and delta 8.2 plasmids were gifts from Dr Dong at Tulane University. Bak, Bax and non-target control (NTC) shRNA lentiviral vectors were purchased from Sigma-Aldrich (St. Louis, MO, USA). Lentivirus production and transduction were carried out as previously described.[12, 13] Briefly, TLA-HEK293T cells were transfected with pMD-VSV-G, delta 8.2, and lentiviral shRNA constructs using Lipofectamine and Plus reagents (Thermo Fisher Scientific) according to the manufacturer's instructions. Virus-containing culture medium was harvested 48 h post-transfection. Cells were transduced for 12 hours using 1 mL of virus supernatant and 4 μg of polybrene and then cultured for an additional 48 h prior to selection with puromycin.

Chromatin fractionation

Chromatin fractionation was carried out as previously described.[5]

Alkaline comet assay

Following treatment with CX-5461, AZD6738, both, or neither for 8-12 hours, U937 (8 hours) and CTS (12 hours) cells were subjected to alkaline comet assay as previously described.[12] Slides were stained with SYBR Gold (Thermo Fisher Scientific), and then imaged on an Olympus BX-40 microscope equipped with a DP72 microscope camera and Olympus cellSens Dimension software (Olympus America Inc., Center Valley, PA, USA). Approximately 50 comets per gel were scored using CometScore (TriTek Corp, Sumerduck, VA, USA).

MTT Assays

MTT (3-[4,5-dimethyl-thiazol-2-yl]-2,5-diphenyltetrazoliumbromide, Sigma-Aldrich) assays in the primary AML patient samples were performed as previously described.[10] Briefly, AML cell lines were treated with variable concentrations of CX-5461, AZD6738, both, or neither, for 72 hours. Cells were then lysed using 10% SDS in 10 mM HCL. IC_{50} values were calculated as the drug concentrations necessary to inhibit 50% growth compared to vehicle control-treated cells. The IC_{50} values are means of duplicates from one experiment due to limited sample. Standard isobologram analysis was performed to determine the extent and direction of anti-leukemic interactions. The IC_{50} values of each drug are plotted on the axes; the solid line represents the additive effect, whereas the points represent the IC_{50} values. Points falling below the line indicate synergistic effect, whereas those above the line indicate antagonistic effect. Patient sample selection was based on availability.

Statistical Analysis

Differences in apoptosis and %DNA in the tail between treated (either individually or combined) and untreated cells were compared via pair-wise two-sample t-test. Differences in IC_{50}s (*TP53*-WT vs. *TP53*-MT and AML vs. healthy controls) were calculated using the Mann-Whitney *U*-test. Statistical analyses were performed using GraphPad Prism 5.0. Error bars represent ± standard error of the mean (SEM); significance level was set at $p < 0.05$.

Data availability

The data supporting the findings of this study are available from the corresponding author upon reasonable request.

References

1. Niu X *et al.* Acute myeloid leukemia cells harboring MLL fusion genes or with the acute promyelocytic leukemia phenotype are sensitive to the Bcl-2-selective inhibitor ABT-199. *Leukemia* **28**, 1557-1560 (2014).
2. Su YW *et al.* Targeting PI3K, mTOR, ERK, and Bcl-2 signaling network shows superior antileukemic activity against AML ex vivo. *Biochem. Pharmacol.* **148**, 13-26 (2018).
3. Uphoff CC, Drexler HG. Detection of mycoplasma contaminations. *Methods Mol. Biol.* **290**, 13-23 (2005).
4. Quentmeier H, Zaborski M, Drexler HG. The human bladder carcinoma cell line 5637 constitutively secretes functional cytokines. *Leuk. Res.* **21**, 343-350 (1997).
5. Ma J *et al.* Mechanisms responsible for the synergistic antileukemic interactions between ATR inhibition and cytarabine in acute myeloid leukemia cells. *Sci. Rep.* **7**, (2017).
6. Xie CZ *et al.* Mechanisms of Synergistic Antileukemic Interactions between Valproic Acid and Cytarabine in Pediatric Acute Myeloid Leukemia. *Clin. Cancer Res.* **16**, 5499-5510 (2010).
7. Edwards H *et al.* RUNX1 regulates phosphoinositide 3-kinase/AKT pathway: role in chemotherapy sensitivity in acute megakaryocytic leukemia. *Blood* **114**, 2744-2752 (2009).
8. Chou TC. Theoretical basis, experimental design, and computerized simulation of synergism and antagonism in drug combination studies. *Pharmacol. Rev.* **58**, 621-681 (2006).
9. Luedtke DA *et al.* Inhibition of Mcl-1 enhances cell death induced by the Bcl-2-selective

inhibitor ABT-199 in acute myeloid leukemia cells. *Signal Transduct Target Ther* **2**, 17012 (2017).

10. Schwartz J *et al.* Synergistic anti-leukemic interactions between ABT-199 and panobinostat in acute myeloid leukemia ex vivo. *American Journal of Translational Research* **8**, 3893-3902 (2016).
11. Ge Y *et al.* Differential gene expression, GATA1 target genes, and the chemotherapy sensitivity of Down syndrome megakaryocytic leukemia. *Blood* **107**, 1570-1581 (2006).
12. Xie CZ *et al.* Panobinostat Enhances Cytarabine and Daunorubicin Sensitivities in AML Cells through Suppressing the Expression of BRCA1, CHK1, and Rad51. *PLoS One* **8**, (2013).
13. Niu X *et al.* Binding of Released Bim to Mcl-1 is a Mechanism of Intrinsic Resistance to ABT-199 which can be Overcome by Combination with Daunorubicin or Cytarabine in AML Cells. *Clin. Cancer Res.* **22**, 4440-4451 (2016).

Figure S1

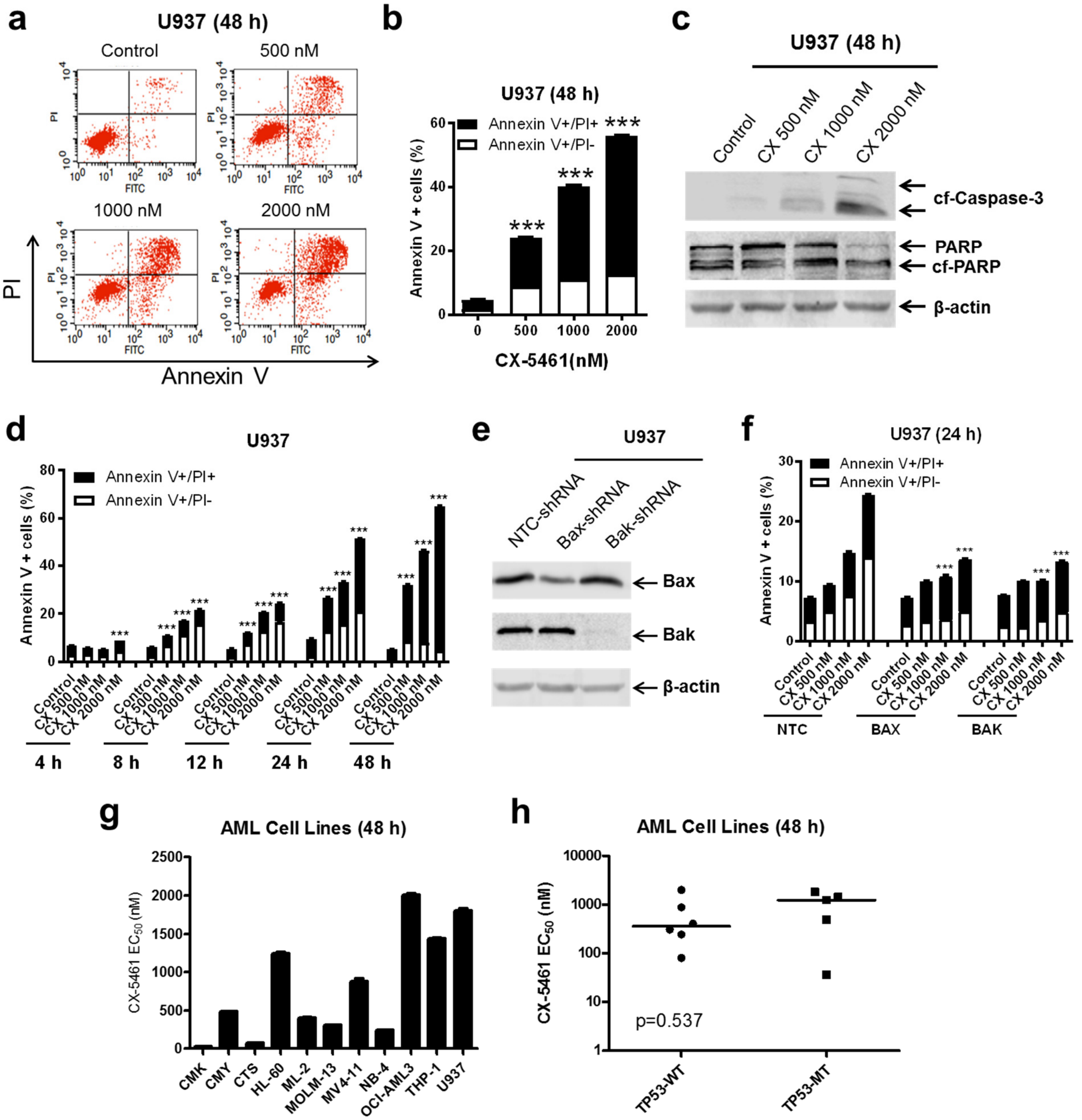

Figure. S1.

CX-4561 induces apoptosis in AML cells. (**a&b**) The AML cell line U937 was treated with CX-5461 (CX) at concentrations of 0 nM (control), 500 nM, 1000 nM, and 2000 nM for 48 hours, and apoptotic induction was measured by Annexin V-FITC/PI staining and flow cytometric analyses; *** indicates p<0.001 (pairwise two sample t-test). (**c**) Whole cell lysates of U937 cells treated with CX-5461 for 48 hours underwent Western blotting and were probed with antibodies, as indicated. cf-caspase 3 indicates cleaved caspase 3 and cf-PARP indicates cleaved PARP. (**d**) U937 cells were treated with CX-5461 for up to 48 hours at the specified concentrations and

apoptotic induction was measured by Annexin V-FITC/PI staining and flow cytometry analyses; *** indicates $p<0.001$ (pairwise two sample t-test). (**e**&**f**) Lentiviral shRNA knockdown of Bax and Bak were performed, with confirmation of knockdown demonstrated via Western blotting of the aforementioned proteins as indicated (panel **e**). Annexin V/PI staining and flow cytometric analyses of CX-5461-induced apoptosis is shown in panel **f**. *** indicates $p<0.001$ (pairwise two sample t-test). (**g**&**h**) AML cell lines were treated with variable concentrations of CX-5461 for 48 h. Annexin V/PI staining and flow cytometry analysis was performed and CX-5461 EC_{50}s were calculated (panel **g**). Comparison of CX-5461 EC_{50} for *TP53*-WT (*TP53*-wild-type; CTS, ML-2, MOLM-13, MV4-11, OCI-AML3, and NB-4) versus *TP53*-MT (*TP53*-mutated; CMK, CMY, HL-60, THP-1, and U937) is shown in panel **h**. The horizontal line indicates the median.

Figure S2

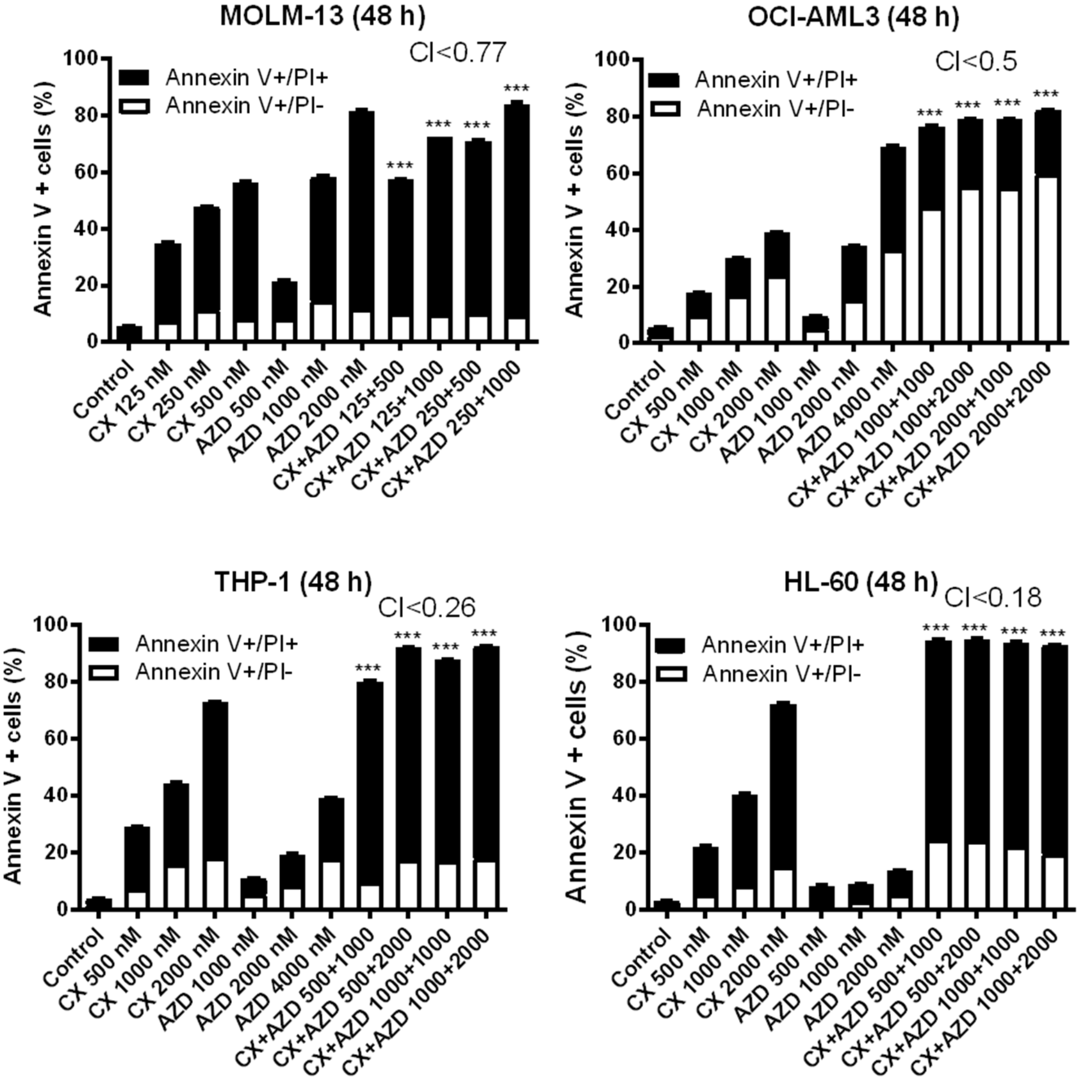

Figure. S2.

Synergistic induction of apoptosis via combined CX-5461 and AZD6738 in AML cell lines. The AML cell lines MOLM-13, OCI-AML3, THP-1, and HL-60 were treated with variable concentrations of CX-5461 (CX) or AZD6738 (AZD) alone or in combination, for 48 hours and then subjected to Annexin V-FITC/PI staining and flow cytometry analyses. *** indicates $p<0.001$ (pairwise two sample t-test). Using CompuSyn software, the combination index (CI) values of this pairing were calculated, where CI=1, <1, and >1 are indicative of additive, synergistic, or antagonistic effect, respectively.

Figure S3

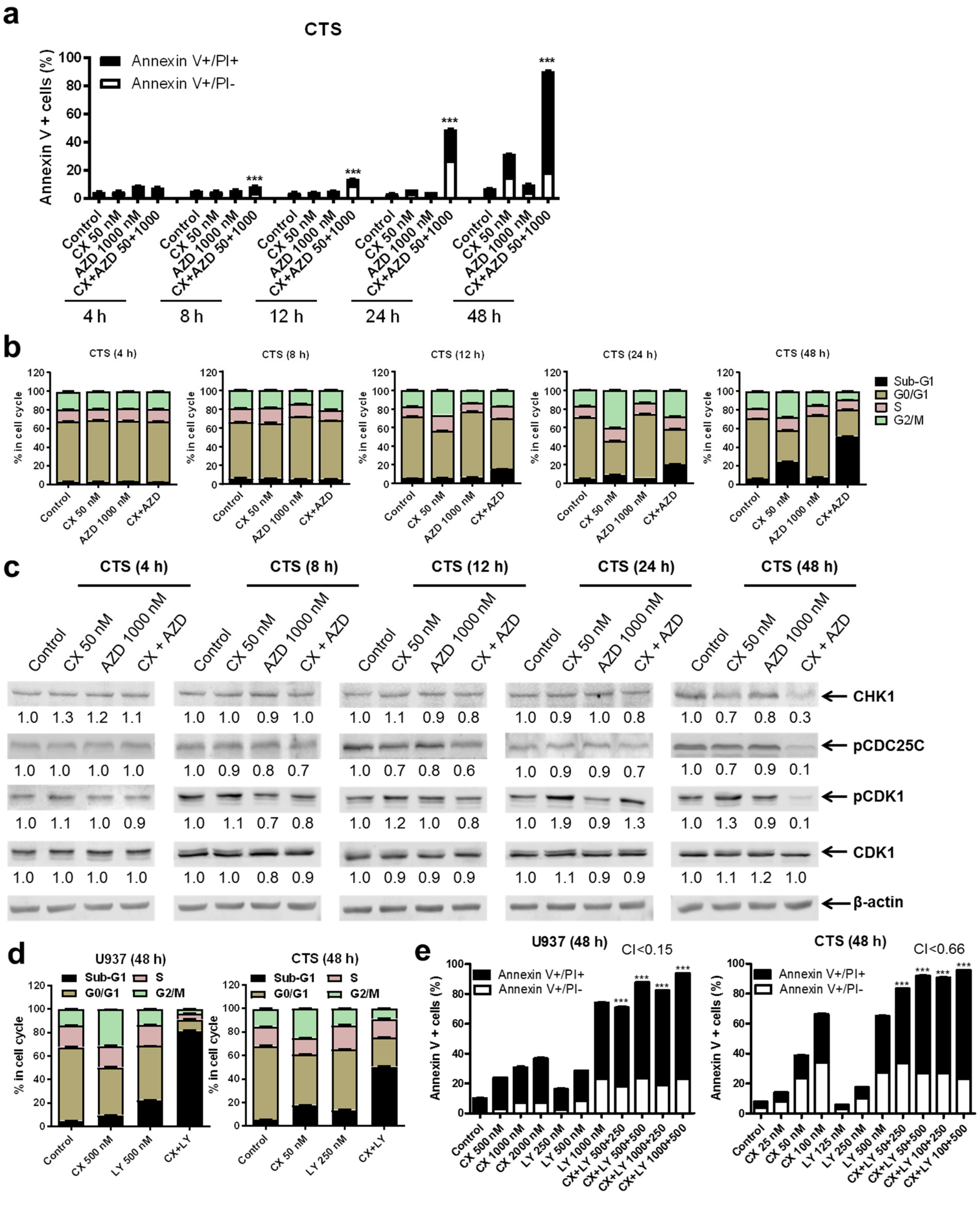

Figure. S3.

G2/M cell cycle checkpoint activation induced by CX-5461 is abolished by AZD6738. (**a**) CTS cells were treated with CX-5461 (CX) or AZD6738 (AZD) alone or in combination, for up to 48

hours and then subjected to Annexin V-FITC/PI staining and flow cytometry analyses; *** indicates p<0.001 (pairwise two sample t-test). (**b**) Cell cycle progression was assessed by propidium iodide (PI) staining and flow cytometry analyses following treatment with CX-5461 or AZD6738, alone or in combination, at concentrations as indicated, for up to 48 hours. (**c**) CTS cells were treated as described in A, whole cell lysates underwent Western blotting and were probed with antibodies as indicated; densitometry fold changes were normalized to β-actin and are displayed below each blot. (**d&e**) Cell cycle progression (panel **d**) and Annexin V-FITC/PI staining with flow cytometry analyses (panel **e**) were performed in CTS and U937 cells treated with CX-5461 or LY2603618 (LY), alone or in combination, at concentrations as indicated, for 48 hours. *** indicates p<0.001 (pairwise two sample t-test). Combination index (CI) of this pairing was calculated using CompuSyn software, where CI=1, <1, and >1 are indicative of additive, synergistic, or antagonistic effect, respectively.

Figure S4

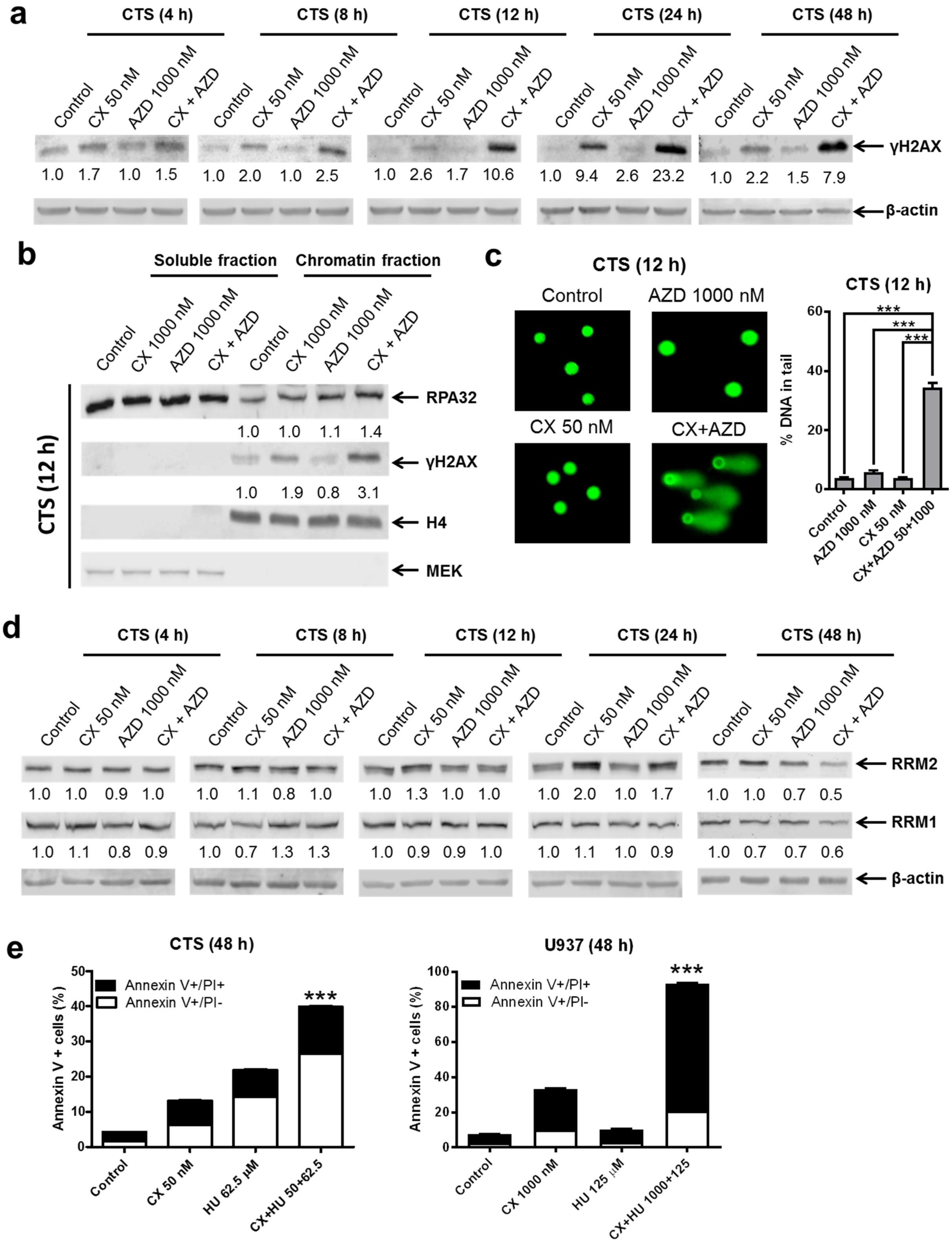
a
CTS (4 h)
CTS (8 h)
CTS (12 h)
CTS (24 h)
CTS (48 h)
Control
CX 50 nM
AZD 1000 nM
CX + AZD
γH2AX
1.0 1.7 1.0 1.5
1.0 2.0 1.0 2.5
1.0 2.6 1.7 10.6
1.0 9.4 2.6 23.2
1.0 2.2 1.5 7.9
β-actin
b
Soluble fraction
Chromatin fraction
Control
CX 1000 nM
AZD 1000 nM
CX + AZD
CTS (12 h)
RPA32
1.0 1.0 1.1 1.4
γH2AX
1.0 1.9 0.8 3.1
H4
MEK
c
CTS (12 h)
Control
AZD 1000 nM
CX 50 nM
CX+AZD
% DNA in tail
***
CX+AZD 50+1000
d
RRM2
1.0 1.0 0.9 1.0
1.0 1.1 0.8 1.0
1.0 1.3 1.0 1.0
1.0 2.0 1.0 1.7
1.0 1.0 0.7 0.5
RRM1
1.0 1.1 0.8 0.9
1.0 0.7 1.3 1.3
1.0 0.9 0.9 1.0
1.0 1.1 1.0 0.9
1.0 0.7 0.7 0.6
β-actin
e
CTS (48 h)
U937 (48 h)
Annexin V+/PI+
Annexin V+/PI-
Annexin V + cells (%)
Control
CX 50 nM
HU 62.5 μM
CX+HU 50+62.5
CX 1000 nM
HU 125 μM
CX+HU 1000+125

Figure. S4.

CX-5461 and AZD6738 cooperate in inducing DNA damage, replication stress in AML cell lines. (**a**) CTS cells were treated with CX-5461 (CX) or AZD6738 (AZD), alone or in combination, at concentrations as indicated, for up to 48 hours and then subjected to Western blotting and were probed with antibodies to γH2AX; densitometry fold changes were normalized to β-actin and are displayed below each blot. (**b**) Levels of RPA32 and γH2AX bound to chromatin and in the soluble fractions of U937 or CTS cells treated with CX-5461 and AZD6738 were analyzed by Western blot. Densitometry fold changes were normalized to histone H4 (H4) and are shown below the corresponding blotting. (**c**) CTS cells were treated with CX-5461 or AZD6738 alone or in combination for 12 hours, and then underwent alkaline comet analyses. Representative visualizations are shown. Results are graphed as the median percentage of DNA in each comet 'tail', following quadruplicate replication, ± SEM. (**d**) CTS were treated with CX-5461 or AZD6738, alone or in combination, at concentrations as indicated, for up to 48 hours and then subjected to Western blotting and were probed with antibodies to ribonucleotide reductase's M1 subunit (RRM1) or M2 subunit (RRM2), as indicated. Densitometry fold changes were normalized to β-actin and are displayed below each blot. (**e**) CTS and U937 cells were treated with CX-5461 and hydroxyurea (HU), alone or in combination, for 48 hours, and then underwent Annexin V-FITC/PI staining and flow cytometry analyses to assess apoptotic induction; *** indicates $p<0.001$ (pairwise two sample t-test).

Figure S5

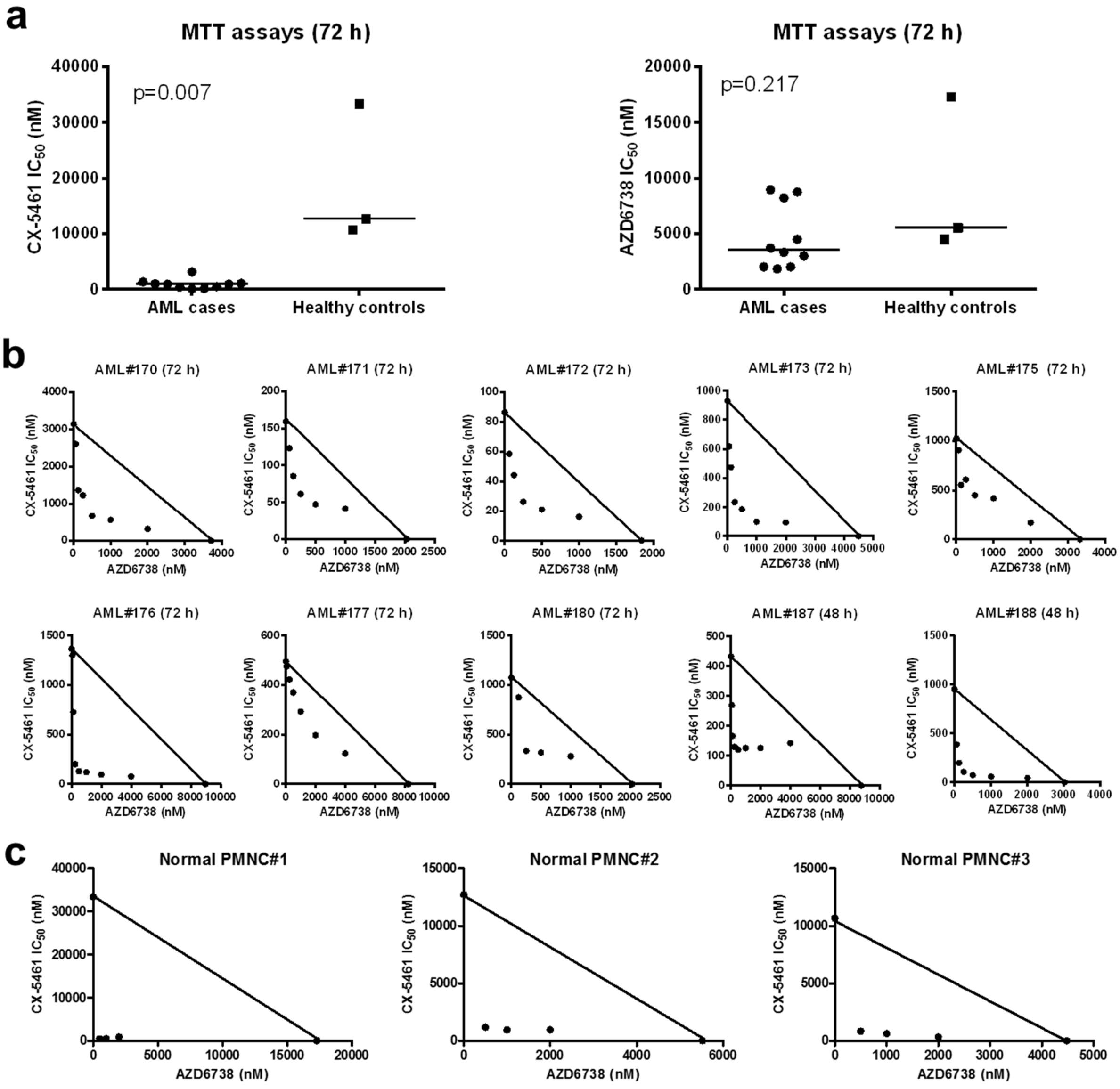

Figure. S5.

Antileukemic activity of CX-5461 and AZD6738 in primary AML patient samples and in normal peripheral blood mononuclear cells (PBMCs). Primary AML patient samples and healthy controls were treated with variable concentrations of CX-5461 or AZD6738, alone or in combination for 72 hours, and subsequently underwent MTT assay assessment. IC50 values displayed are means of duplicates from one experiment due to limited sample; horizontal lines indicate the median (panel **a**). Isobolograms were generated based upon treatment of 10 primary AML patient samples (panel **b**) and 3 healthy PBMC samples (panel **c**).

Table S1.
Patient characteristics of primary AML patient samples

Patients	Gender	Age (year)	Disease status	FAB subtype	Cytogenetics	Blast purity (%)	Gene mutation
AML#166	Male	57	newly diagnosed	M5	46, XY	66	DNMT3A,TET2, NPM-1
AML#170	Female	50	newly diagnosed	M4	46, XX, t(3; 3)(q21; q26)	72.0	SF3B1
AML#171	Female	59	newly diagnosed	M2	47, XX, +8	60.5	FLT3-ITD, DNMT3A
AML#172	Female	54	newly diagnosed	M2	46, XX	58.5	CEBPAdm, IDH2, N-RAS
AML#173	Female	66	newly diagnosed	M2	46, XX	89.5	FLT3-ITD, DNMT3A
AML#175	Male	4	newly diagnosed	M2	46, XY	56.5	
AML#176	Male	35	newly diagnosed	M2	46, XY, t(2;14)(q37;q24)	85	GATA2, c-Kit
AML#177	Male	5	newly diagnosed	M2	46, XY	90	MLL-AF9
AML#180	Male	68	Relapsed	M5	46, XY	96.50%	Flt3-ITD, NPM1, DNMT3A
AML#187	Male	50	Newly diagnosed	M4	47, XY, +8	96.50%	FLT3-ITD
AML#188	Female	55	Newly diagnosed	M3	46, XX,t(15;17)(q22;q21)	93.00%	FLT3-ITD, SH2B3
AML#195	Male	7	Newly diagnosed		46, XY	87%	
AML#198	Female	44	Newly diagnosed	M2	46, XX, t(8;21)(q22;q22)	68%	ASXL1, K-RAS, N-RAS
